# Baseline Inflammatory Biomarkers and Disease Burden for Predicting Response to Stapokibart in CRSwNP

**DOI:** 10.3390/diagnostics16132127

**Published:** 2026-07-07

**Authors:** Yuzhe Hao, Xiangning Cheng, Yuxuan Liu, Shazhou Li, Bingyue Huo, Ziyi Long, Qianxue Hu, Tianjian Xie, Lijun Du, Bo Liu, Xuan Jiao, Shan Chen, Tao Zhou, Liuqing Zhou, Yue Zhou, Jianjun Chen

**Affiliations:** 1 Department of Otolaryngology-Head and Neck Surgery, Union Hospital, Tongji Medical College, Huazhong University of Science and Technology, Wuhan 430022, China; 2 Department of Otolaryngology-Head and Neck Surgery, The First Affiliated Hospital of Shihezi University, Shihezi 832000, China

**Keywords:** chronic rhinosinusitis with nasal polyps, biologics, biomarker, stapokibart, anti-IL-4Rα, complete blood count, efficacy prediction

## Abstract

**Background**: Stapokibart is a novel biologic for chronic rhinosinusitis with nasal polyps (CRSwNP). We aimed to identify baseline biomarkers predicting early (4-week) and mid-term (16-week) responses to stapokibart in CRSwNP. **Methods:** A total of 57 patients were prospectively enrolled. Baseline clinical data and complete blood count (CBC) parameters were collected, and derived inflammatory indices were calculated. Patients were classified as responders or non-responders at week 4 and 16 based on achieving either a ≥8.9-point reduction in SNOT-22 or a ≥1-point decrease in Nasal Polyp Score (NPS). **Results:** Stapokibart significantly improved SNOT-22, VAS, and NPS at both week 4 and week 16 (all *p* < 0.001). At week 4, 80.7% achieved an early response. Responders showed significantly higher baseline eosinophil count and eosinophil percentage and lower neutrophil-to-eosinophil ratio (N/E) (all *p* < 0.05). Univariate analysis identified N/E, comorbid asthma, eosinophil count, and aggregate index of systemic inflammation (AISI) as predictors of early response (all *p* < 0.05). Multivariate analysis identified N/E as an independent predictor (OR = 0.943, *p* = 0.011; AUC = 0.756). At week 16, 75.4% of patients achieved a mid-term response. Responders had significantly higher baseline SNOT-22 scores and NPS (*p* < 0.05). Multivariate analysis showed that baseline NPS and SNOT-22 scores were independently associated with mid-term response, and their combined model showed good predictive performance (AUC = 0.832, 95% CI: 0.716–0.948). **Conclusions:** Peripheral blood inflammatory biomarkers, particularly N/E, may predict early response to stapokibart in CRSwNP, whereas mid-term response appears more strongly associated with baseline disease severity. These findings support biomarker-driven stratification for individualized treatment strategies in CRSwNP.

## 1. Introduction

Chronic rhinosinusitis with nasal polyps (CRSwNP) is a common airway disease characterized by persistent chronic inflammation of the nasal and sinonasal mucosa. It is clinically manifested by severe nasal obstruction, olfactory dysfunction, facial pressure or pain, and sleep disturbance, all of which markedly impair patients’ quality of life [1,2,3,4,5,6]. The prevalence of CRSwNP in the general population is approximately 2–4% [5,7,8], and it accounts for about one-third of patients with chronic rhinosinusitis [9]. Epidemiological studies have further shown that approximately 75% and 50% of patients with CRSwNP have comorbid allergic rhinitis (AR) and asthma, respectively [10]. Current standard treatments include intranasal or systemic corticosteroids and endoscopic sinus surgery. However, some patients exhibit corticosteroid resistance or dependence, and the recurrence rate after surgery ranges from 20% to 60% within 18 months to 4 years [6]. These patients often require repeated surgeries and long-term medication, indicating substantial unmet clinical needs [2,4,11,12,13,14,15,16], which impose considerable distress on patients and a heavy socioeconomic burden.

With increasing understanding of the immunopathogenesis of CRSwNP, the key role of type 2 inflammation in disease development and progression has been widely recognized [4,6,17]. Key type 2 cytokines, such as IL-4 and IL-13, mediate eosinophil infiltration, goblet cell hyperplasia, and tissue remodeling, and are considered core drivers of the formation, progression, and persistence of CRSwNP [17,18]. Therefore, biologics targeting the IL-4/IL-13 pathway have provided a new therapeutic option for CRSwNP [6,18,19,20,21,22,23,24]. Dupilumab, a classic fully human monoclonal antibody against IL-4 receptor α (IL-4Rα), can simultaneously block IL-4 and IL-13 signaling. Multiple global clinical trials have demonstrated that dupilumab significantly reduces nasal polyp size, improves nasal symptoms, and decreases the need for surgical intervention, with a favorable safety profile [19,20,23,25,26]. Stapokibart (CM310) is a novel humanized monoclonal antibody targeting IL-4Rα. Its phase III clinical study also demonstrated significant therapeutic efficacy in CRSwNP, showing reductions in nasal polyp size. During 52 weeks of maintenance treatment, nearly 90% of patients achieved a reduction of at least 50% in nasal polyp volume, accompanied by alleviation of nasal symptom severity [6,21,27].

Although anti-IL-4Rα monoclonal antibodies have demonstrated clear efficacy in the overall population of patients with CRSwNP, 20–30% of patients still show poor treatment responses in clinical practice [28,29]. Therefore, early identification of patients who are likely to benefit from treatment and avoidance of ineffective therapy have become key issues in achieving precision treatment for CRSwNP [6,24]. Peripheral blood complete blood count (CBC) parameters and their derived inflammatory markers [30,31], such as eosinophil count, neutrophil-to-lymphocyte ratio (NLR), platelet-to-lymphocyte ratio (PLR), systemic immune-inflammation index (SII), systemic inflammation response index (SIRI), and aggregate index of systemic inflammation (AISI), have been explored for predicting postoperative recurrence and evaluating the efficacy of biologics in CRSwNP [16,28,32,33]. However, existing findings remain inconsistent. Some studies have shown that NLR or PLR can predict the response to dupilumab treatment [32], whereas others have found no significant association [28]. In particular, studies on predictors of early and mid-term response to stapokibart in patients with CRSwNP remain lacking.

Therefore, we conducted a single-center, prospective, single-arm study involving patients with CRSwNP treated with stapokibart. This study aimed to evaluate the efficacy of stapokibart at week 4 (early response) and week 16 (mid-term response), and to systematically analyze the predictive value of baseline clinical characteristics and CBC-derived inflammatory indices for early and mid-term treatment responses. Particular attention was paid to differences in predictors between early and mid-term responses, with the goal of providing evidence to support the selection of patients most likely to benefit from treatment and the optimization of individualized therapeutic strategies in clinical practice.

## 2. Materials and Methods

### 2.1. Study Design

This was a single-center, prospective, single-arm, observational study designed to evaluate the short-term and mid-term efficacy of stapokibart in patients with CRSwNP, and to analyze the effects and predictive value of relevant baseline factors on treatment outcomes. The study population included patients with CRSwNP who received stapokibart treatment in the Department of Otolaryngology–Head and Neck Surgery, Union Hospital, Tongji Medical College, Huazhong University of Science and Technology, from July to December 2025. The period from January to June 2025 involved preliminary patient screening and clinical data collection preparation prior to the official commencement of the study. All study procedures complied with the principles of the Declaration of Helsinki and were approved by the Ethics Committee of Union Hospital, Tongji Medical College, Huazhong University of Science and Technology (approval No. UHCT22444).

### 2.2. Patient Screening

Patients with primary CRSwNP were screened and enrolled after providing written informed consent. Patient screening was performed strictly according to the following criteria.

Inclusion criteria:

(1) A clinical diagnosis of CRSwNP according to the Chinese Guideline for the Diagnosis and Treatment of Chronic Rhinosinusitis (2024);
(2) Age between 18 and 65 years, regardless of sex;
(3) A history of sinus surgery at least 6 months before screening, or systemic corticosteroid treatment within the previous 2 years, with a washout period of 6 weeks;
(4) Complete baseline clinical data, laboratory test results, and follow-up records.

Exclusion criteria:

(1) Nasal polyps secondary to cystic fibrosis, fungal rhinosinusitis, immunodeficiency, or other secondary causes;
(2) Symptoms of viral infection, fever, or other systemic manifestations within 1 month before enrollment, or confirmed active or acute infection requiring medication;
(3) Pregnancy, lactation, or recent pregnancy plans;
(4) Severe systemic diseases or other conditions considered unsuitable for participation in this study;
(5) Inability to obtain informed consent.

### 2.3. Clinical Protocol

At the initial consultation, patients underwent nasal endoscopy and completed symptom assessments, including the visual analogue scale (VAS) and the 22-item Sino-Nasal Outcome Test (SNOT-22), under the guidance of physicians to evaluate nasal polyp size and symptom severity. At the same time, demographic and clinical characteristics were collected in detail through electronic questionnaires, including age, sex, body mass index (BMI), BMI category, duration of chronic rhinosinusitis, number and history of previous sinus surgeries, comorbid allergic rhinitis, comorbid asthma, and family history of allergic diseases, including allergic rhinitis, chronic rhinosinusitis, allergic asthma, and any allergic disease. Blood samples were collected to measure total IgE and CBC parameters, including leukocytes, platelets, neutrophils, lymphocytes, monocytes, eosinophils, basophils, and their corresponding percentages. CBC-derived inflammatory indices were calculated, including the NLR, PLR, SII, SIRI, AISI, lymphocyte-to-monocyte ratio (LMR), platelet-to-white blood cell ratio (PWR), neutrophil-to-eosinophil ratio (N/E), and eosinophil-to-lymphocyte ratio (E/L).

Patients enrolled in this study received an initial subcutaneous injection of 300 mg stapokibart, followed by 300 mg subcutaneous injections every 2 weeks at our hospital throughout the study period. During follow-up, patients completed VAS and SNOT-22 assessments under the guidance of physicians. Nasal endoscopy was performed at week 4 and week 16. Based on the endoscopic findings, the nasal polyp score (NPS) was jointly assessed by two experienced otolaryngologists. The total bilateral NPS ranged from 0 to 8, with each side scored as follows: 0, no polyps; 1, small polyps confined to the middle meatus; 2, polyps extending beyond the middle meatus; 3, large polyps reaching the lower border of the inferior turbinate; and 4, polyps causing complete nasal obstruction [34]. Concomitant medications during treatment were determined by clinicians according to each patient’s condition. The follow-up cutoff date was 31 December 2025.

### 2.4. Study Outcomes

The efficacy endpoint of this study was treatment response to stapokibart. With reference to previous studies [25,26,28,35,36,37], treatment response was defined as achieving at least one clinically meaningful improvement criterion: (1) a decrease of ≥1 point in the NPS; (2) a decrease of ≥8.9 points in the SNOT-22 score, which represents the minimal clinically important difference (MCID). The 4-week and 16-week follow-up visits were defined as the time points for evaluating early and mid-term responses, respectively [25]. Patients were classified into responder and non-responder groups according to whether they achieved a treatment response, and intergroup comparisons were then performed.

### 2.5. Statistical Analysis

All statistical analyses were performed using R software, version 4.4.3, and SPSS, version 27.0. The normality of continuous variables was first assessed using the Shapiro–Wilk test. Normally distributed variables are presented as mean ± standard deviation (mean ± SD), whereas non-normally distributed variables are presented as median with interquartile range [median (P25, P75)]. Categorical variables are expressed as frequencies and percentages [n (%)]. The paired Wilcoxon signed-rank test was used to compare changes in scores from baseline to week 4 and week 16. For intergroup comparisons, normally distributed continuous variables were analyzed using the independent-samples *t*-test, whereas non-normally distributed variables were analyzed using the Wilcoxon rank-sum test. Categorical variables were analyzed using the chi-square test or Fisher’s exact test, as appropriate. Binary logistic regression analysis was performed to evaluate the predictive value of baseline variables for early and mid-term responses. Variables with *p* < 0.10 in univariate analysis were selected for inclusion in the multivariate model. Due to the limited sample size and number of events, multivariable models included no more than two variables to avoid overfitting. Results are presented as odds ratios (ORs) and 95% confidence intervals (CIs). Receiver operating characteristic (ROC) curves were generated, and the area under the curve (AUC) with 95% CI was calculated. All tests were two-sided, and *p* < 0.05 was considered statistically significant.

## 3. Results

### 3.1. Demographic and Baseline Characteristics

A total of 57 eligible patients with CRSwNP who received stapokibart treatment and completed the 16-week follow-up were included in this study. Among them, 31 patients were male (54%) and 26 were female (46%). The median age was 44.0 years (36.0, 54.0), and the median disease duration was 4.0 years (2.0, 10.0). Twenty-six patients (46%) had a history of sinus surgery, 38 (67%) had comorbid allergic rhinitis, and 23 (40%) had comorbid asthma. At baseline, the SNOT-22 score was 38.8 ± 18.0, the median VAS score was 70.0 (50.0, 80.0), and the median NPS was 4.0 (3.0, 4.0). The median peripheral blood levels of total IgE, platelets, lymphocytes, eosinophils, and basophils were 114.5 (52.5, 216.0) kU/L, 233.0 (205.0, 266.0) × 10^9^/L, 2.0 (1.7, 2.4) × 10^9^/L, 0.4 (0.3, 0.5) × 10^9^/L, and 0.0 (0.0, 0.1) × 10^9^/L, respectively. The mean leukocyte, neutrophil, and monocyte counts were 6.8 ± 1.4 × 10^9^/L, 3.9 ± 1.2 × 10^9^/L, and 0.4 ± 0.1 × 10^9^/L, respectively. Baseline clinical and laboratory characteristics are shown in Table 1.

### 3.2. Overall Treatment Outcomes

**Figure 1:**
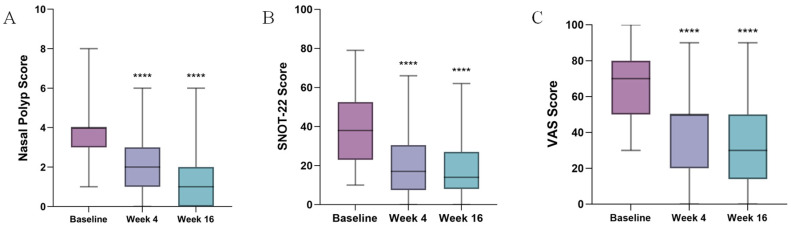
Box plots showing changes in NPS (**A**), SNOT-22 (**B**), and VAS (**C**) before and after treatment. **** *p* < 0.0001 versus baseline, indicating a statistically significant difference.

Compared with baseline, symptom scores and NPS were significantly improved in all patients at both 4 and 16 weeks after treatment (all *p* < 0.001). The NPS decreased from 4.0 (3.0, 4.0) at baseline to 2.0 (1.0, 3.0) at week 4 and 1.0 (0.0, 2.0) at week 16. The SNOT-22 score decreased from 38.8 ± 18.0 to 21.5 ± 17.8 and 18.7 ± 15.1, respectively. The VAS score decreased from 70.0 (50.0, 80.0) to 50.0 (20.0, 50.0) and 30.0 (15.0, 50.0), respectively. These results indicate that stapokibart was associated with favorable improvements in symptoms and nasal polyp burden in this cohort (Figure 1).

### 3.3. Comparison of Baseline Indicators Between Early Responders and Non-Responders

Among all patients, 80.7% (46/57) showed a favorable response to stapokibart at week 4 and were classified as responders, whereas the remaining 19.3% (11/57) did not show a treatment response and were classified as non-responders. Table 2 shows the comparison of baseline clinical characteristics and laboratory indicators between early responders and non-responders. Statistical analysis revealed significant differences between the two groups in age, comorbid asthma, VAS score, eosinophil count, eosinophil percentage, basophil percentage, and N/E (*p* < 0.05). At baseline, patients who were younger, had comorbid asthma, higher VAS scores, higher eosinophil counts and percentages, higher basophil percentages, and lower N/E were more likely to achieve an early response.

### 3.4. Comparison of Baseline Indicators Between Mid-Term Responders and Non-Responders

At week 16, 75.4% (43/57) of patients showed a favorable treatment response and were classified as responders, whereas 24.6% (14/57) were classified as non-responders. Table 3 shows the comparison of baseline clinical characteristics and laboratory indicators between mid-term responders and non-responders. The results suggested that only baseline SNOT-22 and NPS differed significantly between the two groups (*p* < 0.05). Specifically, responders had higher baseline SNOT-22 scores than non-responders (42.0 ± 18.2 vs. 29.2 ± 14.2, *p* = 0.018), as well as higher baseline NPS [4.0 (3.0, 5.0) vs. 2.5 (2.0, 4.0), *p* = 0.021].

### 3.5. Binary Logistic Regression Analysis for Predicting Treatment Response

To evaluate the ability of baseline clinical and laboratory data to predict the response to stapokibart treatment, early response and mid-term response were used as dependent variables, and univariate and multivariate logistic regression analyses were performed separately.

**Figure 2:**
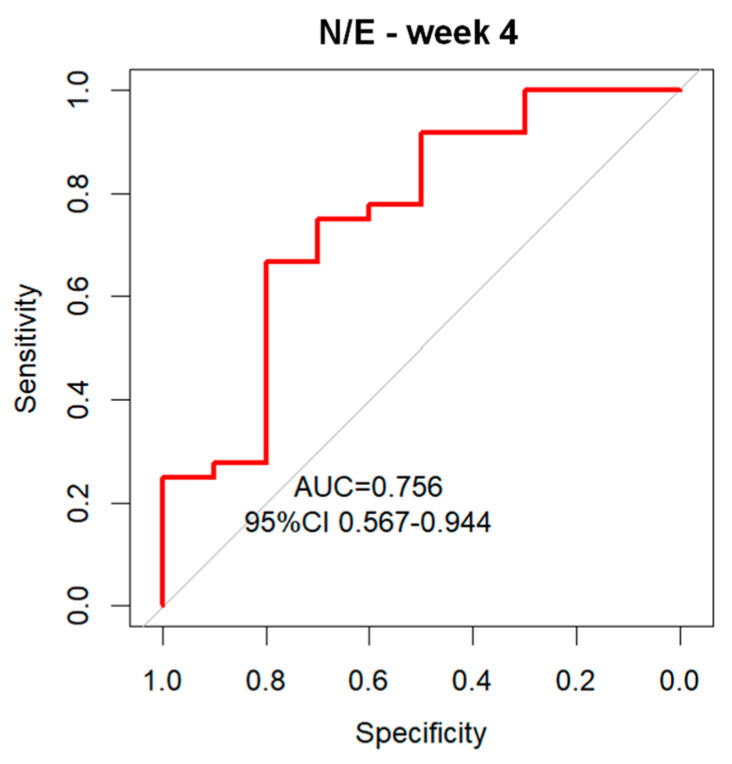
ROC curve of N/E for predicting early response at week 4.

For the prediction of early response, univariate analysis suggested that N/E (OR = 0.928, 95% CI: 0.870–0.975, *p* = 0.008), eosinophil count (OR = 7.460, 95% CI: 1.390–39.965, *p* = 0.019), comorbid asthma (OR = 6.430, 95% CI: 1.337–63.000, *p* = 0.018), and AISI (OR = 0.994, 95% CI: 0.988–0.999, *p* = 0.042) showed potential predictive value. After including N/E and comorbid asthma in the multivariate regression model, N/E was identified as an independent factor associated with early treatment response (OR = 0.943, 95% CI: 0.887–0.987, *p* = 0.011), with an AUC of 0.756 (95% CI: 0.567–0.944) (Figure 2). In contrast, comorbid asthma did not reach statistical significance (OR = 2.756, 95% CI: 0.475–28.8, *p* = 0.272) (Table 4).

In the univariate analysis of baseline data for mid-term response, baseline NPS (OR = 1.882, 95% CI: 1.164–3.358, *p* = 0.018) and baseline SNOT-22 score (OR = 1.047, 95% CI: 1.008–1.095, *p* = 0.027) were significantly associated with mid-term response. Other variables, including age, sex, history of sinus surgery, comorbid asthma, complete blood count parameters, and derived inflammatory indices, did not show clear predictive value in the univariate logistic regression analysis (all *p* > 0.10). Both variables were then included in the multivariate regression model, which indicated that baseline NPS and SNOT-22 score were independent predictors of mid-term response (NPS: OR = 2.020, 95% CI: 1.213–3.799, *p* = 0.015; SNOT-22: OR = 1.057, 95% CI: 1.012–1.115, *p* = 0.023) (Table 5).

**Figure 3:**
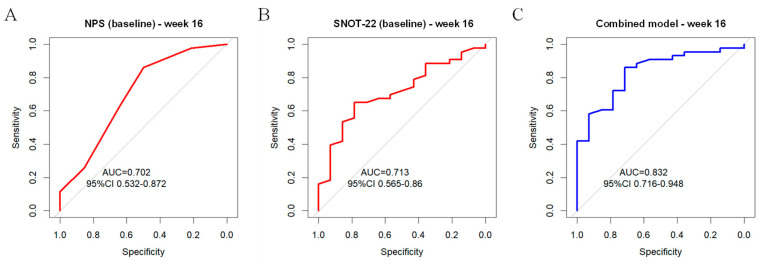
ROC curves of baseline NPS (**A**), SNOT-22 (**B**), and their combined model (**C**) for predicting mid-term response at week 16.

To further evaluate the discriminative ability of each indicator and the combined model for mid-term response, ROC curves were generated, and the AUC was calculated (Figure 3). The results indicated that baseline NPS alone yielded an AUC of 0.702 (95% CI: 0.532–0.872), while baseline SNOT-22 alone yielded an AUC of 0.713 (95% CI: 0.565–0.860), indicating that both had certain predictive value. The combined model consisting of baseline NPS and SNOT-22 showed a higher AUC of 0.832 (95% CI: 0.716–0.948), suggesting that it may have better discriminative ability than either indicator alone.

## 4. Discussion

This study evaluated the short-term and mid-term efficacy of stapokibart in 57 patients with CRSwNP and systematically analyzed the predictive value of baseline clinical characteristics and peripheral blood CBC-derived inflammatory indices for treatment response. The results indicated that SNOT-22, VAS, and NPS scores were significantly improved from baseline after 4 and 16 weeks of treatment. The response rates were 80.7% at week 4 and 75.4% at week 16, indicating favorable therapeutic benefits, which were generally consistent with previous clinical studies of stapokibart [21,27]. In this study, the response rate at week 16 was slightly lower than that at week 4. This may be related to the limited sample size. Other possible reasons include a plateau phase after rapid suppression of inflammation during early targeted therapy, individual differences in treatment response, and the influence of potential comorbidities. Future studies with larger sample sizes are needed to further analyze the trajectories of sustained response, delayed response, and loss of response. At present, clinical studies on stapokibart for the treatment of CRSwNP remain limited. This study provides preliminary real-world evidence for the early efficacy and response prediction of stapokibart in patients with CRSwNP, suggesting that it may improve symptoms and nasal polyp burden during the early stage of treatment.

In this study, early response was significantly associated with several indicators reflecting type 2 inflammation in the univariate analysis, including eosinophil count, AISI, N/E, and comorbid asthma. This suggests that patients with a higher systemic type 2 inflammatory burden may be more likely to show a rapid response to stapokibart, which is consistent with the mechanism by which anti-IL-4Rα monoclonal antibodies rapidly suppress type 2 inflammation by blocking IL-4/IL-13 signaling [6,18,38]. Owing to the limited sample size, only N/E and comorbid asthma were included in the multivariate regression model. N/E was identified as an independent factor associated with early treatment response (OR = 0.943, *p* = 0.011), whereas comorbid asthma did not reach statistical significance. These indicators require further validation in large-scale, multicenter studies.

In contrast to early response, the independent predictors of mid-term response were baseline NPS and SNOT-22 score, rather than peripheral blood inflammatory markers. This finding suggests that the mid-term efficacy of anti-IL-4Rα therapy may be more closely related to disease burden at enrollment than to the level of systemic type 2 inflammation. The combined model incorporating baseline NPS and SNOT-22 showed an AUC of 0.832 (95% CI: 0.716–0.948), which was higher than that of baseline NPS or SNOT-22 alone (NPS: AUC = 0.702; SNOT-22: AUC = 0.713), suggesting that the combined model may have better discriminative ability. Further external validation and optimization of this model in large-scale, multicenter cohorts are needed to facilitate its clinical application. Habenbacher et al. [28] reported that baseline eosinophil count may be a potential biomarker for nasal polyp reduction in patients with CRSwNP treated with dupilumab, whereas NLR, PLR, and other peripheral blood inflammatory markers showed no significant associations. Notably, in the present study, none of the blood-derived inflammatory markers, including total IgE, eosinophil count, NLR, PLR, SII, SIRI, and AISI, entered the prediction model for mid-term response. This may be because the mid-term benefit of stapokibart is influenced by multiple factors, such as local inflammatory status, the degree of tissue remodeling, and nasal polyp burden, while baseline NPS and SNOT-22 may comprehensively reflect these complex pathophysiological features. In addition, because treatment response in this study was defined using absolute decreases in outcome scores, patients with higher baseline scores had greater room for improvement. Therefore, the predictive value of baseline NPS and SNOT-22 may partly reflect differences in baseline score levels and improvement potential, and this finding should be further validated in larger and external cohorts.

Stapokibart is a recently developed humanized monoclonal antibody targeting IL-4Rα. It can effectively block the binding of IL-4 and IL-13 to IL-4Rα, thereby inhibiting their roles in type 2 inflammatory responses [38,39]. Stapokibart has been clinically used for the treatment of atopic dermatitis [40,41] CRSwNP [21,27], and moderate-to-severe seasonal allergic rhinitis [42], showing favorable therapeutic effects across these diseases. An important finding of this study is that the predictors of response to stapokibart differed between week 4 and week 16. Early response at week 4 was mainly associated with indicators reflecting systemic type 2 inflammation, such as N/E and eosinophil count. In contrast, mid-term response at week 16 was more closely associated with baseline disease burden, including SNOT-22 and NPS. This suggests that the initial treatment response may primarily reflect the inhibitory effect of stapokibart on type 2 inflammation, whereas mid-term efficacy may be influenced by a broader range of factors. Previous studies have reported that approximately 40–60% of patients with CRSwNP respond poorly to biologic therapy [24], highlighting the importance of selecting suitable patients to improve the therapeutic efficacy of biologics. In the real-world use of stapokibart, peripheral blood inflammatory markers may help predict early response and rapidly identify patients who are likely to benefit, whereas baseline disease severity may be more suitable for predicting mid-term efficacy and guiding longer-term treatment decisions. These findings provide new insights into precision treatment for CRSwNP. Moreover, because CRSwNP is a local mucosal inflammatory disease, additional local biomarkers (e.g., nasal smear, nasal secretion analysis, nasal polyp biopsy, and nasal nitric oxide) may help refine patient stratification. Future studies should integrate local biomarker analyses to optimize precision treatment and response prediction for stapokibart.

This study has several potential limitations. First, this was a real-world, single-arm observational study without a placebo or active comparator group; therefore, the effects of the natural disease course and placebo response could not be excluded. In addition, concomitant treatments were not fully standardized, which may have influenced the efficacy assessment. Future studies should standardize and document concomitant therapies to reduce potential bias and validate the observed efficacy outcomes. Second, the study population was derived from a single center, and the sample size was limited, which may affect the stability and generalizability of the findings. In the future, these indicators must be validated in larger external cohorts to confirm their clinical value. Third, treatment response was defined only by NPS and SNOT-22, without incorporating other clinical outcome measures, such as nasal congestion score (NC) or loss of smell score (LoS), which may affect the robustness of the results. The response definition used in this study was based on achieving clinically meaningful improvement in either objective or patient-reported outcomes, consistent with previous biologic studies in CRSwNP. However, this definition may have overestimated the response rate compared with stricter composite endpoints requiring simultaneous improvement in both domains. Future studies should consider more stringent composite endpoints or stratified response definitions. Fourth, because treatment response was defined using absolute reductions in outcome scores, patients with higher baseline symptom burden inherently had greater potential for improvement. Therefore, the observed association between baseline severity and treatment response may partly reflect regression-to-the-mean effects or mathematical coupling. Future studies should evaluate percentage-based improvement metrics and adjusted longitudinal models to further validate these findings. Fifth, the follow-up period was limited to 16 weeks and was insufficient to evaluate the long-term efficacy of stapokibart; longer follow-up is needed to confirm the durability of treatment benefits. Sixth, peripheral blood parameters were collected only at baseline, without dynamic monitoring during treatment. Future large-scale, multicenter, prospective studies with standardized long-term follow-up and dynamic CBC monitoring are needed to further identify valuable baseline clinical characteristics and biomarkers that can predict the efficacy of stapokibart in patients with CRSwNP.

## 5. Conclusions

Stapokibart rapidly and persistently improved symptoms and nasal polyp burden in patients with CRSwNP at week 4 and week 16. Factors associated with treatment response may vary over time: early response was mainly related to eosinophil level and N/E, whereas mid-term response was more closely associated with baseline disease severity, including baseline NPS and SNOT-22 scores. The combined model based on baseline NPS and SNOT-22 showed good discriminative ability. Further validation and optimization of this prediction model in large-scale, multicenter cohorts are warranted.

## Figures and Tables

**Table 1:** Baseline clinical and laboratory characteristics.

Characteristic	N = 57 ^1^
Age	44.0 (36.0, 54.0)
Sex	
Male	31 (54%)
Female	26 (46%)
BMI	23.4 (21.1, 25.7)
BMI category	
Class 1 (<18.5)	2 (3.5%)
Class 2 (18.5–23.9)	32 (56%)
Class 3 (24.0–27.9)	16 (28%)
Class 4 (≥28.0)	7 (12%)
Duration of CRSwNP	4.0 (2.0, 10.0)
Number of previous sinus surgeries	0.0 (0.0, 1.0)
History of sinus surgery	
No	31 (54%)
Yes	26 (46%)
Comorbid allergic rhinitis	
No	19 (33%)
Yes	38 (67%)
Comorbid asthma	
No	34 (60%)
Yes	23 (40%)
Family history of allergic rhinitis	
No	37 (65%)
Yes	20 (35%)
Family history of CRSwNP	
No	50 (88%)
Yes	7 (12%)
Family history of allergic asthma	
No	49 (86%)
Yes	8 (14%)
SNOT-22	38.8 ± 18.0
VAS	70.0 (50.0, 80.0)
NPS	4.0 (3.0, 4.0)
Total IgE (kU/L)	114.5 (52.5, 216.0)
Leukocytes (×10^9^/L)	6.8 ± 1.4
Platelets (×10^9^/L)	233.0 (205.0, 266.0)
Neutrophils (×10^9^/L)	3.9 ± 1.2
Lymphocytes (×10^9^/L)	2.0 (1.7, 2.4)
Monocytes (×10^9^/L)	0.4 ± 0.1
Eosinophils (×10^9^/L)	0.4 (0.3, 0.5)
Basophils (×10^9^/L)	0.0 (0.0, 0.1)
Neutrophil percentage	56.3 ± 8.7
Lymphocyte percentage	30.5 ± 7.2
Eosinophil percentage	5.2 (4.0, 7.0)
Basophil percentage	0.6 (0.3, 0.8)
Monocyte percentage	6.5 ± 1.4
NLR	1.9 (1.4, 2.4)
PLR	124.2 (96.2, 147.3)
SII	461.4 (321.2, 537.3)
SIRI	0.8 (0.6, 1.0)
AISI	196.5 (121.6, 268.6)
LMR	4.9 ± 1.4
PWR	36.2 ± 9.1
N/E	10.8 (8.1, 15.9)
E/L	0.2 (0.1, 0.2)

^1^ Continuous variables are expressed as Mean ± SD or Median (P25, P75), and categorical variables are expressed as n (%).

**Table 2:** Comparison of baseline indicators between early responders and non-responders.

Characteristic	Non-Responders	Responders	*p*-Value ^2^
	N = 11 ^1^	N = 46 ^1^	
Age	51.2 ± 7.2	44.4 ± 15.9	0.027
Sex			0.5
Male	7 (64%)	24 (52%)	
Female	4 (36%)	22 (48%)	
BMI	23.6 ± 2.7	23.6 ± 3.7	>0.9
Duration of CRSwNP	7.0 (2.0, 14.0)	4.0 (1.5, 8.0)	0.4
Number of previous sinus surgeries	1.0 (0.0, 1.0)	0.0 (0.0, 1.0)	0.13
History of sinus surgery			0.2
No	4 (36%)	27 (59%)	
Yes	7 (64%)	19 (41%)	
Comorbid allergic rhinitis			>0.9
No	4 (36%)	15 (33%)	
Yes	7 (64%)	31 (67%)	
Comorbid asthma			0.037
No	10 (91%)	24 (52%)	
Yes	1 (9.1%)	22 (48%)	
Family history of allergic rhinitis			0.2
No	5 (45%)	32 (70%)	
Yes	6 (55%)	14 (30%)	
Family history of CRSwNP			0.3
No	11 (100%)	39 (85%)	
Yes	0 (0%)	7 (15%)	
Family history of allergic asthma			>0.9
No	10 (91%)	39 (85%)	
Yes	1 (9.1%)	7 (15%)	
SNOT-22	34.6 ± 16.4	39.8 ± 18.5	0.4
VAS	50.0 (50.0, 70.0)	70.0 (50.0, 85.0)	0.046
NPS	3.0 (2.0, 4.0)	4.0 (3.0, 4.0)	0.2
Total IgE (kU/L)	74.8 (43.2, 150.0)	121.5 (76.9, 249.0)	0.12
Leukocytes (×10^9^/L)	7.1 ± 1.6	6.7 ± 1.4	0.6
Platelets (×10^9^/L)	241.5 (227.0, 259.0)	227.0 (197.5, 272.0)	0.7
Neutrophils (×10^9^/L)	4.4 ± 1.4	3.7 ± 1.1	0.2
Lymphocytes (×10^9^/L)	1.7 (1.6, 2.0)	2.1 (1.8, 2.4)	0.2
Monocytes (×10^9^/L)	0.5 ± 0.1	0.4 ± 0.1	0.4
Eosinophils (×10^9^/L)	0.3 (0.1, 0.4)	0.4 (0.3, 0.6)	0.005
Basophils (×10^9^/L)	0.0 (0.0, 0.0)	0.0 (0.0, 0.1)	0.074
Neutrophil percentage	60.8 ± 8.9	55.1 ± 8.3	0.091
Lymphocyte percentage	28.4 ± 8.3	31.1 ± 6.9	0.3
Eosinophil percentage	3.5 (1.2, 5.0)	6.0 (4.2, 7.0)	0.039
Basophil percentage	0.4 (0.3, 0.5)	0.7 (0.4, 0.8)	0.028
Monocyte percentage	6.4 ± 1.3	6.5 ± 1.4	0.7
NLR	2.2 (1.7, 3.0)	1.8 (1.4, 2.3)	0.2
PLR	128.3 (92.9, 156.6)	122.5 (97.9, 142.1)	0.6
SII	516.7 (321.2, 682.6)	435.2 (326.0, 529.4)	0.2
SIRI	1.0 (0.7, 1.7)	0.8 (0.5, 1.0)	0.2
AISI	232.4 (173.6, 443.7)	193.3 (120.4, 251.7)	0.2
LMR	4.7 ± 1.8	5.0 ± 1.3	0.5
PWR	35.6 ± 8.9	36.3 ± 9.3	0.8
N/E	19.5 (12.8, 49.9)	9.4 (7.3, 13.9)	0.013
E/L	0.1 (0.1, 0.2)	0.2 (0.1, 0.3)	0.072

^1^ Continuous variables are expressed as Mean ± SD or Median (P25, P75), and categorical variables are expressed as n (%). ^2^ Wilcoxon rank sum test; Pearson’s Chi-squared test; Fisher’s exact test; Wilcoxon rank sum exact test.

**Table 3:** Comparison of baseline indicators between mid-term responders and non-responders.

Characteristic	Non-Responders	Responders	*p*-Value ^2^
	N = 14 ^1^	N = 43 ^1^	
Age	46.2 ± 10.0	45.6 ± 16.2	0.4
Sex			0.4
Male	9 (64%)	22 (51%)	
Female	5 (36%)	21 (49%)	
BMI	23.6 ± 2.1	23.6 ± 3.9	0.8
Duration of CRSwNP	6.5 (1.0, 10.0)	4.0 (2.0, 8.0)	0.7
Number of previous sinus surgeries	0.5 (0.0, 1.0)	0.0 (0.0, 1.0)	0.4
History of sinus surgery			0.7
No	7 (50%)	24 (56%)	
Yes	7 (50%)	19 (44%)	
Comorbid allergic rhinitis			0.8
No	4 (29%)	15 (35%)	
Yes	10 (71%)	28 (65%)	
Comorbid asthma			0.1
No	11 (79%)	23 (53%)	
Yes	3 (21%)	20 (47%)	
Family history of allergic rhinitis			0.7
No	10 (71%)	27 (63%)	
Yes	4 (29%)	16 (37%)	
Family history of CRSwNP			0.2
No	14 (100%)	36 (84%)	
Yes	0 (0%)	7 (16%)	
Family history of allergic asthma			>0.9
No	12 (86%)	37 (86%)	
Yes	2 (14%)	6 (14%)	
SNOT-22	29.2 ± 14.2	42.0 ± 18.2	0.018
VAS	70.0 (50.0, 80.0)	70.0 (50.0, 80.0)	>0.9
NPS	2.5 (2.0, 4.0)	4.0 (3.0, 5.0)	0.021
Total IgE (kU/L)	83.0 (43.2, 150.0)	125.0 (76.9, 281.0)	0.095
Leukocytes (×10^9^/L)	6.5 ± 1.3	6.9 ± 1.5	0.3
Platelets (×10^9^/L)	227.0 (210.0, 248.0)	244.0 (205.0, 276.0)	0.5
Neutrophils (×10^9^/L)	3.9 ± 1.4	3.9 ± 1.1	0.7
Lymphocytes (×10^9^/L)	1.8 (1.6, 2.2)	2.1 (1.8, 2.4)	0.2
Monocytes (×10^9^/L)	0.4 ± 0.1	0.4 ± 0.1	0.9
Eosinophils (×10^9^/L)	0.3 (0.2, 0.4)	0.4 (0.3, 0.5)	0.13
Basophils (×10^9^/L)	0.0 (0.0, 0.1)	0.0 (0.0, 0.1)	0.5
Neutrophil percentage	57.8 ± 11.0	55.8 ± 7.7	0.4
Lymphocyte percentage	29.9 ± 8.4	30.7 ± 6.8	0.7
Eosinophil percentage	4.5 (2.4, 6.8)	5.8 (4.3, 7.0)	0.2
Basophil percentage	0.5 (0.3, 0.7)	0.6 (0.4, 0.8)	0.4
Monocyte percentage	6.5 ± 1.2	6.5 ± 1.4	0.8
NLR	2.0 (1.4, 2.8)	1.9 (1.4, 2.3)	0.6
PLR	127.9 (92.9, 148.5)	123.2 (99.6, 138.8)	0.8
SII	496.0 (290.5, 647.6)	446.2 (353.9, 532.1)	0.8
SIRI	0.9 (0.6, 1.2)	0.8 (0.6, 1.0)	0.8
AISI	192.4 (106.2, 284.9)	199.0 (134.5, 253.6)	>0.9
LMR	4.7 ± 1.6	4.9 ± 1.4	0.7
PWR	36.3 ± 8.1	36.1 ± 9.6	0.9
N/E	14.0 (8.1, 24.7)	9.9 (8.2, 14.2)	0.3
E/L	0.2 (0.1, 0.2)	0.2 (0.1, 0.3)	0.2

^1^ Continuous variables are expressed as Mean ± SD or Median (P25, P75), and categorical variables are expressed as n (%). ^2^ Wilcoxon rank sum test; Pearson’s Chi-squared test; Fisher’s exact test; Wilcoxon rank sum exact test.

**Table 4:** Univariate and multivariate logistic regression analyses of early treatment response.

Baseline Indicators	Univariate Analysis			Multivariate Analysis		
	OR	95% CI	*p* Value	OR	95% CI	*p* Value
N/E	0.928	0.870–0.975	0.008	0.943	0.887–0.987	0.011
Comorbid asthma	6.430	1.337–63.000	0.018	2.756	0.475–28.821	0.272
Eosinophils (×10^9^/L)	7.460	1.390–39.965	0.019			
AISI	0.994	0.988–0.999	0.042			

**Table 5:** Univariate and multivariate logistic regression analyses of mid-term treatment response.

Baseline Indicators	Univariate Analysis			Multivariate Analysis		
	OR	95% CI	*p* Value	OR	95% CI	*p* Value
NPS	1.882	1.164–3.358	0.018	2.020	1.213–3.799	0.015
SNOT-22	1.047	1.008–1.095	0.027	1.057	1.012–1.115	0.023

## Data Availability

The original contributions presented in the study are included in the article, further inquiries can be directed to the corresponding author.

## Abbreviations

The following abbreviations are used in this manuscript:

AISI	Aggregate index of systemic inflammation
AR	Allergic rhinitis
AUC	Area under the curve
BMI	Body mass index
CBC	Complete blood count
CI	Confidence interval
CRSwNP	Chronic rhinosinusitis with nasal polyps
E/L	Eosinophil-to-lymphocyte ratio
IgE	Immunoglobulin E
IL-4	Interleukin-4
IL-13	Interleukin-13
IL-4Rα	Interleukin-4 receptor alpha
LMR	Lymphocyte-to-monocyte ratio
LoS	Loss of smell score
MCID	Minimal clinically important difference
N/E	Neutrophil-to-eosinophil ratio
NC	Nasal congestion score
NLR	Neutrophil-to-lymphocyte ratio
NPS	Nasal polyp score
OR	Odds ratio
PLR	Platelet-to-lymphocyte ratio
PWR	Platelet-to-white blood cell ratio
ROC	Receiver operating characteristic
SD	Standard deviation
SII	Systemic immune-inflammation index
SIRI	Systemic inflammation response index
SNOT-22	22-item Sino-Nasal Outcome Test
SPSS	Statistical Package for the Social Sciences
VAS	Visual analogue scale

## References

[1] Ryu G., Kim D.W. (2020). Th2 inflammatory responses in the development of nasal polyps and chronic rhinosinusitis. Curr. Opin. Allergy Clin. Immunol..

[2] Wang H., Pan L., Liu Z. (2019). Neutrophils as a Protagonist and Target in Chronic Rhinosinusitis. Clin. Exp. Otorhinolaryngol..

[3] Zhang C., Wang H., Hu L., Zhang Q., Chen J., Shi L., Song X., Liu J., Xue K., Wang J. (2024). Lipocalin-2 promotes neutrophilic inflammation in nasal polyps and its value as biomarker. Allergol. Int..

[4] Fokkens W.J., Lund V.J., Hopkins C., Hellings P.W., Kern R., Reitsma S., Toppila-Salmi S., Bernal-Sprekelsen M., Mullol J., Alobid I. (2020). European Position Paper on Rhinosinusitis and Nasal Polyps 2020. Rhinology.

[5] Fokkens W.J., De Corso E., Backer V., Bernal-Sprekelsen M., Bjermer L., von Buchwald C., Chaker A., Diamant Z., Gevaert P., Han J. (2024). EPOS2020/EUFOREA expert opinion on defining disease states and therapeutic goals in CRSwNP. Rhinology.

[6] Xian M., Yan B., Song X., Chen J., Tang J., Jiang Y., Wan L., Liu W., Xue J., Cao Z. (2025). Chinese Position Paper on Biologic Therapy for Chronic Rhinosinusitis With Nasal Polyps. Allergy.

[7] Chen S., Zhou A., Emmanuel B., Thomas K., Guiang H. (2020). Systematic literature review of the epidemiology and clinical burden of chronic rhinosinusitis with nasal polyposis. Curr. Med. Res. Opin..

[8] Han J.K., Bachert C., Fokkens W., Desrosiers M., Wagenmann M., Lee S.E., Smith S.G., Martin N., Mayer B., Yancey S.W. (2021). Mepolizumab for chronic rhinosinusitis with nasal polyps (SYNAPSE): A randomised, double-blind, placebo-controlled, phase 3 trial. Lancet Respir. Med..

[9] Sedaghat A.R., Kuan E.C., Scadding G.K. (2022). Epidemiology of Chronic Rhinosinusitis: Prevalence and Risk Factors. J. Allergy Clin. Immunol. Pract..

[10] Yao Y., Zeng M., Liu Z. (2022). Revisiting Asian chronic rhinosinusitis in the era of type 2 biologics. Clin. Exp. Allergy.

[11] Riva G., Tavassoli M., Cravero E., Moresco M., Albera A., Canale A., Pecorari G. (2022). Long-term evaluation of nasal polyposis recurrence: A focus on multiple relapses and nasal cytology. Am. J. Otolaryngol..

[12] Shah S.A., Kobayashi M. (2023). Pathogenesis of chronic rhinosinusitis with nasal polyp and a prominent T2 endotype. Heliyon.

[13] Zhang C., Zhang Q., Chen J., Li H., Cheng F., Wang Y., Gao Y., Zhou Y., Shi L., Yang Y. (2024). Neutrophils in nasal polyps exhibit transcriptional adaptation and proinflammatory roles that depend on local polyp milieu. JCI Insight.

[14] Lu H., Lin X.S., Yao D.M., Zhuang Y.Y., Wen G.F., Shi J., Sun Y.Q. (2018). Increased serum amyloid A in nasal polyps is associated with systemic corticosteroid insensitivity in patients with chronic rhinosinusitis with nasal polyps: A pilot study. Eur. Arch. Oto-Rhino-Laryngol..

[15] Chen Y.S., Feng C.Y., Su S.H., Wang Y.H., Yang T.H., Lin C.F. (2025). Recurrence of Chronic Rhinosinusitis with Nasal Polyps After Surgery: Risk Factors, Predictive Models, and Treatment Approaches with a Focus on Western and Asian Differences. Medicina.

[16] Yang Y., Zhu J., Zhang M., Wang Y., Cheng F., Ma W., Li M. (2024). Systemic inflammation response index predicts the postoperative recurrence of chronic rhinosinusitis with nasal polyps: A retrospective study in the Chinese population. Eur. Arch. Oto-Rhino-Laryngol..

[17] Kotas M.E., Patel N.N., Cope E.K., Gurrola J.G., Goldberg A.N., Pletcher S.D., Seibold M.A., Moore C.M., Gordon E.D. (2023). IL-13-associated epithelial remodeling correlates with clinical severity in nasal polyposis. J. Allergy Clin. Immunol..

[18] Chen J., Zhang C., Zhang Q., Cheng F., Wang Y., Xue S., Yang Y., Guo W., Liu J., Xue K. (2025). Targeting IL-4/IL-13 Signaling Pathways in Chronic Rhinosinusitis with Nasal Polyps: From Mechanisms to Therapies. Clin. Rev. Allergy Immunol..

[19] De Corso E., Canonica G.W., Heffler E., Springer M., Grzegorzek T., Viana M., Horváth Z., Mullol J., Gevaert P., Michel J. (2025). Dupilumab versus omalizumab in patients with chronic rhinosinusitis with nasal polyps and coexisting asthma (EVEREST): A multicentre, randomised, double-blind, head-to-head phase 4 trial. Lancet Respir. Med..

[20] Bachert C., Han J.K., Desrosiers M., Hellings P.W., Amin N., Lee S.E., Mullol J., Greos L.S., Bosso J.V., Laidlaw T.M. (2019). Efficacy and safety of dupilumab in patients with severe chronic rhinosinusitis with nasal polyps (LIBERTY NP SINUS-24 and LIBERTY NP SINUS-52): Results from two multicentre, randomised, double-blind, placebo-controlled, parallel-group phase 3 trials. Lancet.

[21] Shen S., Yan B., Wang M., Wu D., Piao Y., Tang J., Yang X., Cao Z., Xue J., Liu W. (2025). Stapokibart for Severe Uncontrolled Chronic Rhinosinusitis With Nasal Polyps: The CROWNS-2 Randomized Clinical Trial. Jama.

[22] Zheng M., Wu D., Piao Y., Tang J., Quan F., Guan B., Yu H., Zhang X., He G., Yang Y. (2025). Efficacy and safety of GR1802 in uncontrolled chronic rhinosinusitis with nasal polyps: Placebo-controlled phase 2 trial. J. Allergy Clin. Immunol..

[23] De Corso E., Pasquini E., Trimarchi M., La Mantia I., Pagella F., Ottaviano G., Garzaro M., Pipolo C., Torretta S., Seccia V. (2023). Dupilumab in the treatment of severe uncontrolled chronic rhinosinusitis with nasal polyps (CRSwNP): A multicentric observational Phase IV real-life study (DUPIREAL). Allergy.

[24] Guo C.L., Liu F.F., Wang D.Y., Liu Z. (2023). Type 2 Biomarkers for the Indication and Response to Biologics in CRSwNP. Curr. Allergy Asthma Rep..

[25] Bachert C., Khan A.H., Fokkens W.J., Hopkins C., Gevaert P., Han J.K., Hellings P.W., Lee S.E., Msihid J., Nash S. (2024). Dupilumab response onset, maintenance, and durability in patients with severe CRSwNP. J. Allergy Clin. Immunol..

[26] Bachert C., Peters A.T., Heffler E., Han J.K., Olze H., Pfaar O., Chuang C.C., Rout R., Attre R., Goga L. (2022). Responder analysis to demonstrate the effect of targeting type 2 inflammatory mechanisms with dupilumab across objective and patient-reported endpoints for patients with severe chronic rhinosinusitis with nasal polyps in the SINUS-24 and SINUS-52 studies. Clin. Exp. Allergy.

[27] Zhang Y., Yan B., Shen S., Song X., Jiang Y., Shi L., Zhao C., Yang Y., Jiang L., Li J. (2023). Efficacy and safety of CM310 in severe eosinophilic chronic rhinosinusitis with nasal polyps (CROWNS-1): A multicentre, randomised, double-blind, placebo-controlled phase 2 clinical trial. EClinicalMedicine.

[28] Habenbacher M., Moser U., Abaira A., Kiss P., Holzmeister C., Pock J., Walla K., Lang A., Andrianakis A. (2024). Investigation of Blood Count-Based Inflammatory Biomarkers as Predictors of Response to Dupilumab Treatment in Patients with Chronic Rhinosinusitis with Nasal Polyps. Pharmaceutics.

[29] Seys S.F., Schneider S., de Kinderen J., Reitsma S., Cavaliere C., Tomazic P.V., Morgenstern C., Mortuaire G., Wagenmann M., Bettio G. (2025). Real-world effectiveness of dupilumab in a European cohort of chronic rhinosinusitis with nasal polyps (CHRINOSOR). J. Allergy Clin. Immunol..

[30] Lampros M., Vlachodimitropoulou L., Voulgaris S., Alexiou G.A. (2026). Correlation of Routine Admission Inflammatory Biomarkers with Individual Traumatic Brain Lesion Types in Mild Traumatic Brain Injury. Biomedicines.

[31] Alci A., Yalcin N., Gokkaya M., Ekin Sari G., Turkmenoglu H.R., Ureyen I., Toptas T. (2025). The Relationship Between Peripheral Inflammatory Markers and High-Grade Cervical Lesions: A Retrospective Cohort Study. Diagnostics.

[32] Brkic F.F., Liu D.T., Rücklinger I., Campion N.J., Bartosik T.J., Vyskocil E., Stanek V., Tu A., Gangl K., Schneider S. (2023). Platelet-to-lymphocyte ratio might predict the response to dupilumab treatment for patients with nasal polyposis. J. Otolaryngol..

[33] Canakci H., Yazici H., Yayman S., Tulaci K.G., Arslan E., Hizli O. (2025). Beyond the CBC: Can Novel Markers Predict Recurrence in Chronic Rhinosinusitis With Nasal Polyposis?. Clin. Otolaryngol..

[34] Gevaert P., De Craemer J., Bachert C., Blauwblomme M., Chaker A., Cingi C., Hellings P.W., Hopkins C., Hox V., Fokkens W.J. (2023). European Academy of Allergy and Clinical Immunology position paper on endoscopic scoring of nasal polyposis. Allergy.

[35] Chuang C.C., Guillemin I., Bachert C., Lee S.E., Hellings P.W., Fokkens W.J., Duverger N., Fan C., Daizadeh N., Amin N. (2022). Dupilumab in CRSwNP: Responder Analysis Using Clinically Meaningful Efficacy Outcome Thresholds. Laryngoscope.

[36] Bachert C., Khan A.H., Hopkins C., Blaiss M.S., Soler Z.M., Nash S., Siddiqui S., Praestgaard A., Deniz Y., Rowe P.J. (2022). Rapid and Continuing Improvements in Nasal Symptoms with Dupilumab in Patients with Severe CRSwNP. J. Asthma Allergy.

[37] Han J.K., Bachert C., Lee S.E., Hopkins C., Heffler E., Hellings P.W., Peters A.T., Kamat S., Whalley D., Qin S. (2022). Estimating Clinically Meaningful Change of Efficacy Outcomes in Inadequately Controlled Chronic Rhinosinusitis with Nasal Polyposis. Laryngoscope.

[38] Liu W., Zhao Y., He Y., Yan X., Yu J., Song Q., Zhang L., Dong B., Xu G., Wang C. (2024). Stapokibart (CM310) targets IL-4Rα for the treatment of type 2 inflammation. iScience.

[39] Shirley M. (2025). Stapokibart: First Approval. Drugs.

[40] Zhao Y., Zhang J., Yang B., Li J., Ding Y., Wu L., Zhang L., Wang J., Zhu X., Zhang F. (2024). Efficacy and safety of CM310 in moderate-to-severe atopic dermatitis: A multicenter, randomized, double-blind, placebo-controlled phase 2b trial. Chin. Med. J..

[41] Zhao Y., Li J.Y., Yang B., Ding Y.F., Wu L.M., Zhang L.T., Wang J.Y., Lu Q.J., Zhang C.L., Zhang F.R. (2024). Long-Term Efficacy and Safety of Stapokibart in Adults with Moderate-to-Severe Atopic Dermatitis: An Open-Label Extension, Nonrandomized Clinical Trial. BioDrugs.

[42] Zhang Y., Li J., Wang M., Li X., Yan B., Liu J., Shi L., Cao Z., Feng Y., Liu W. (2025). Stapokibart for moderate-to-severe seasonal allergic rhinitis: A randomized phase 3 trial. Nat. Med..

